# Assessment of retinal and choroidal microvasculature in night shift medical workers by OCT angiography

**DOI:** 10.1038/s41598-024-62863-w

**Published:** 2024-06-03

**Authors:** Congbi Liang, Yishuang Xu, Qinyun Xia, Di Xiao, Jingsai Gu, Xiangxiang Zhu, Changzheng Chen, Zhen Chen, Dihao Hua

**Affiliations:** Eye Center, Renmin Hospital of Wuhan University, 238 Jiefang Road, Wuchang District, Wuhan, 430060 Hubei China

**Keywords:** Biomarkers, Medical research

## Abstract

This study evaluated retinal and choroidal microvascular changes in night shift medical workers and its correlation with melatonin level. Night shift medical workers (group A, 25 workers) and non-night shift workers (group B, 25 workers) were recruited. The images of macula and optic nerve head were obtained by swept-source OCT-angiography. Vessel density of retina, choriocapillaris (CC), choriocapillaris flow deficit (CC FD), choroidal thickness (CT) and choroidal vascularity index (CVI) were measured. 6-sulfatoxymelatonin concentration was analyzed from the morning urine. CC FD and CVI were significantly decreased and CT was significantly increased in group A (all *P* < 0.05). 6-sulfatoxymelatonin concentration was significantly lower in group A (*P* < 0.05), which was significantly positively correlated with CC FD size (*r* = 0.318, *P* = 0.024) and CVI of the most regions (maximum *r*-value was 0.482, *P* < 0.001), and was significantly negatively associated with CT of all regions (maximum *r*-value was − 0.477, *P* < 0.001). In night shift medical workers, the reduction of melatonin was significantly correlated with CT thickening, CVI reduction and CC FD reduction, which suggested that they might have a higher risk of eye diseases. CC FD could be a sensitive and accurate indicator to reflect CC perfusion.

## Introduction

Night work is becoming common in modern society since the intensification of social production pressure and the refinement of the division of labor. In industrialized countries, 15–20% of the workforce was required to do night shift work, especially in the healthcare sector where the proportion of shift workers was as high as 45%^1^. Night shift work could disrupt circadian rhythms, affecting metabolism and hormone levels^1^, which established risk factors for some systemic diseases, including obesity, diabetes, depression, cancer and cardiovascular disease^2,3^. It was also related to some ophthalmic diseases, such as central serous chorioretinopathy (CSC)^4^. Evidence suggested that retinal microvessels could serve as noninvasive biomarkers to reflect the occurrence and progression of systemic and retinal diseases^5^. Exploring the impact of circadian rhythm changes on ocular microvessels can increase the understanding of eye diseases related to circadian rhythms, and may even have a hint for systemic diseases.

Melatonin was synthesized by the pineal gland and released into the circulation in a circadian rhythm, which production was sensitive to light with being inhibited in daylight and reaching a peak in darkness^6^. In addition, it was proved that the melatonin level decreased in night shift workers^7^. Melatonin could regulate the blood flow of organ vascular bed, including kidney and retina^8,9^. Recent studies showed that melatonin was also considered as a potential therapy of multiple retinal diseases^9,10^, which indicated that it could be involved in the mechanism of their development. Studying the effects of melatonin on the circulation in retina and choroid might help us understand the impact of circadian rhythm disorders on retina and choroid.

Previous research used OCT-angiography (OCTA) to study the changes of choroidal microvessels in normal people within 24 h and found that these parameters demonstrated significant diurnal variations^11^. These rhythmical variations could be regulated by rhythmical hormones. Thus, the circadian rhythm disorders might impact the ocular microvasculature and might even induce retinal diseases. In this study, we explored the changes of retinal and choroidal microvessels in regular night shift medical workers by swept-source OCTA (SS-OCTA), and analyzed choriocapillaris flow deficit (CC FD) which has been confirmed as a sensitive and new indicator of occurrence and progress in many ophthalmic diseases^12,13^. In addition, as an important hormone in circadian rhythm disorders, the relationship between melatonin and ocular microvasculature was also analyzed in our study.

## Methods

### Subjects

This cross-sectional study was conducted at Renmin Hospital of Wuhan University. Night shift medical workers (group A) and non-night shift workers (group B) were recruited. This study was approved by Clinical Research Ethics Committee of Renmin Hospital of Wuhan University (WDRM2022-K106), and all researches and data collection complied with the Declaration of Helsinki. All participants signed written informed consent before entering the study.

This study included medical workers who had night shift work for more than two years, with a frequency of at least once a week. The night shifts operated from 5 pm to 8am the next day. The exclusion criteria for all subjects were as follows: (1) age < 18 years, (2) refractive error >  ± 3 diopters, (3) systemic diseases (diabetes, hypertension, and hyperthyroidism), (4) drinking coffee or alcohol 12 h before the study, (5) BMI < 18.5 kg/m^2^ or BMI > 23.9 kg/m^2^, or(6) any history of ocular disease (glaucoma, diabetic retinopathy and uveitis) or surgeries.

All participants underwent a complete ophthalmic examination, including best-corrected visual acuity, intraocular pressure (IOP) measurements with a noncontact tonometer, slit lamp examination and SS-OCTA. Randomly select one eye of participants. Subjects were interviewed and information was collected on social demographics and lifestyle (coffee and alcohol consumption, smoking and medication).

### SS-OCTA imaging

**Figure 1:**
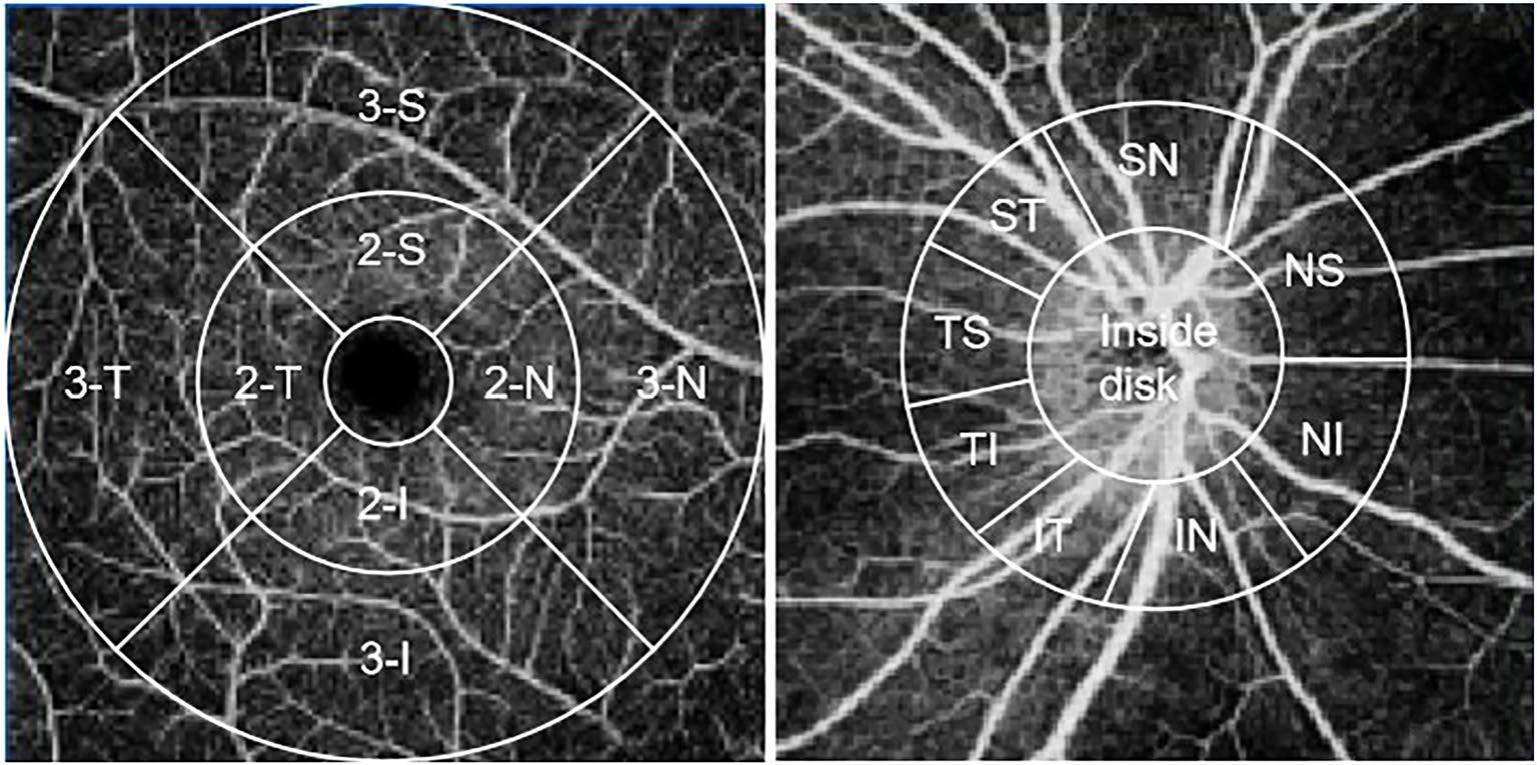
Nine zones of the macula (left) and optic disc (right). *I* inferior, *T* temporal, *S* superior, *N* nasal, *NS* nasal superior, *NI* nasal inferior, *IN* inferior nasal, *IT* inferior tempo, *TI* tempo inferior, *TS* tempo superior, *ST* superior tempo, *SN* superior nasal.

All participants underwent OCTA imaging with a macula and an optic nerve head 6 × 6 mm angiography model by a SS-OCTA (VG100; SVision Imaging, Ltd., Luoyang, China). Three concentric images with diameters of 1, 3 and 6 mm were formed with the fovea as the center of the tested eye. Concentric circles with diameters of 1–3 mm and 3–6 mm were defined as the parafoveal area (area 2) and perifoveal area (area 3), respectively. The parafoveal and perifoveal area were respectively subdivided into four parts: superior (S), inferior (I), nasal (N) and temporal (T) (Fig. 1). The ring area with a width of 2 mm centered on the optic disc was taken as the area of measurement around the optic disc, which was divided into nine subfields: inside-disc, nasal inferior (NI), inferior nasal (IN), inferior tempo (IT), tempo inferior (TI), tempo superior (TS), superior tempo (ST), superior nasal (SN) and nasal superior (NS) (Fig. 1). The equipment's built-in software was used to measure the vessel density of superficial capillary plexus (SCP), deep capillary plexus (DCP), radial peripapillary capillary (RPC) and choriocapillaris (CC), choroidal vascularity index (CVI) and choroidal thickness (CT).

The segmentation of the retina layers were manually checked and corrected by the same ophthalmologist. The slab between the nerve fiber layer and the 1/3 ganglion cell complex was defined as SCP. DCP was defined as the slab between the 1/2 inner nuclear layer and the outer plexiform layer. CC was defined as a 20 μm slab below the Bruch’s membrane. The distance between the Bruch’s membrane and the choroid-sclera interface was defined as CT. The CVI was defined as the proportion of the luminal area to the total choroidal area. The quantification of CT and CVI was calculated by the built-in algorithm through the B-scan of 6 × 6 mm OCTA images in the macular region. Motion artifacts were minimized by the instrument's built-in eye movement tracker system. All OCTA images were measured by the well-trained examiners daily at 8 am to standardize the diurnal effect across all subjects. Images with low image quality (< 7/10), poor image clarity, and blurred were excluded.

### CC FD analysis

**Figure 2:**
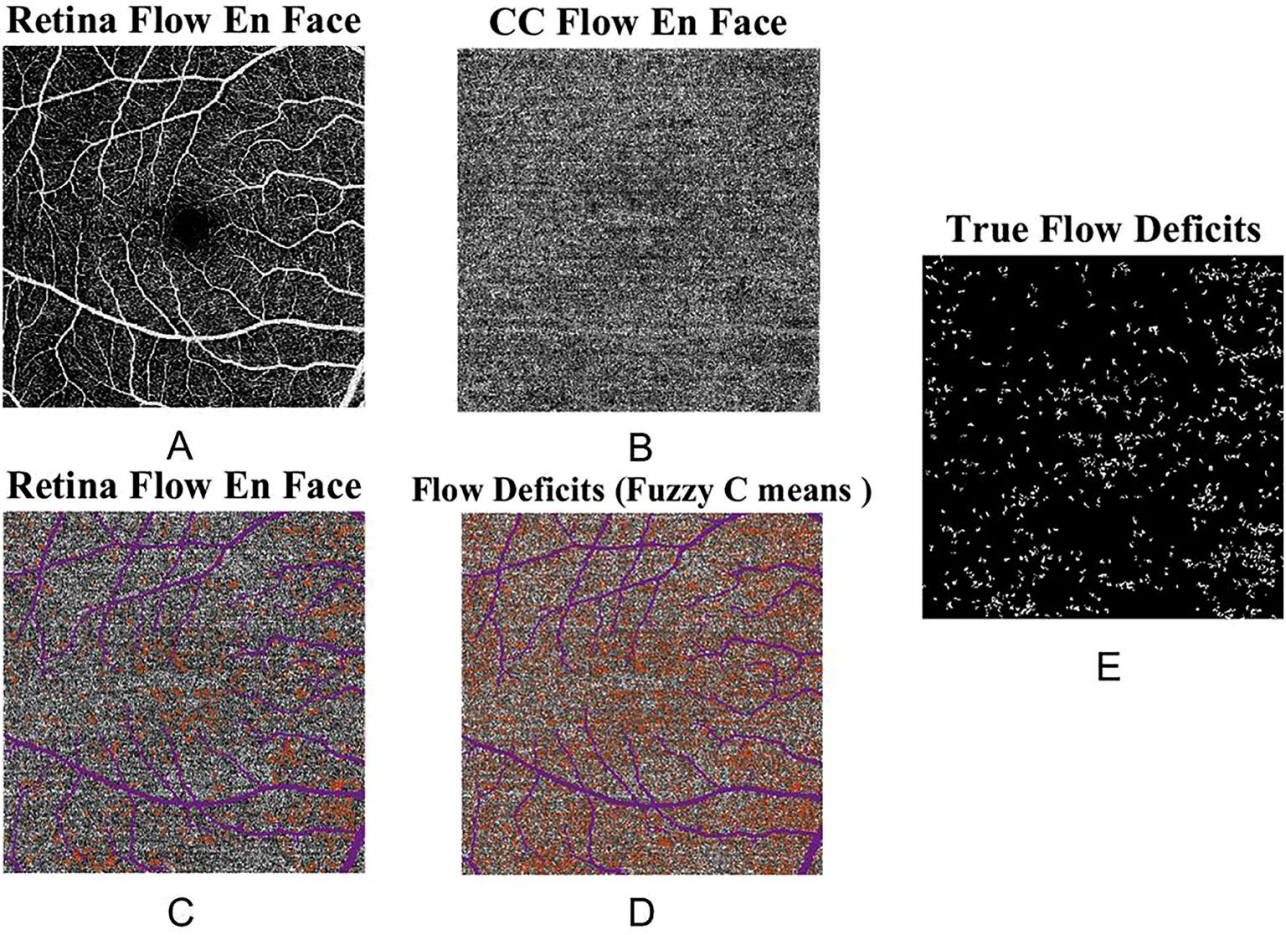
An example of extracting choriocapillaris flow deficit (CC FD) from swept-source OCT-angiography (SS-OCTA) images of the CC. (**A**) and (**B**) represent the en face SS-OCTA images of the retina and CC. The corresponding retinal large vessels are shown in purple (**C**). The FDs are shown as orange, and the projection area of the retinal large vessels is shown as purple (**D**). (**E**) shows the true FDs after excluding the region underneath the retinal large vessels and the flow voids < 24 µm in diameter.

CC FD was defined as the area with flow below a fixed threshold within the CC. The analysis of the CC FD was performed using the CC slab. A binary mask of the larger blood vessels from the retinal images was generated by applying a fixed threshold binarization algorithm. This binary mask was then overlaid on the CC en face images to remove the influence of shadow of the larger blood vessels from the calculation of the CC indices. Fuzzy C means^14^ algorithm was used to segment vasculature and CC FD from the CC image. After CC FD segmentation, FDs with an equivalent diameter smaller than 24 µm were excluded from further analysis. Two quantitative indices were calculated: mean FD size and FD density. Mean FD size (µm^2^) was the averaged size of all the detected FDs in the entire region. FD density was defined as the percentage of the image area occupied by the CC FDs to the area of the entire region in the clustered CC image. An example of the en face SS-OCTA CC image and the corresponding FD segmentation was shown in Fig. 2.

### Melatonin detection

6-sulfatoxymelatonin (aMT6s) was a urinary metabolite of rapid metabolism and excretion of serum melatonin and was a reliable reflection of melatonin^15^. In our study, participants' morning urine was collected at 8 am. A total of 50 urine samples were collected, each in a 15 mL plastic tube, and the date of each collection was marked on the plastic tube. Urine aMT6s was performed immediately after specimen collection.

Urinary aMT6s was assayed in duplicate by using 6-hydroxymelatoin sulfate ELISA Kit (EU3120, FineTest, Wuhan Fine Biotech Co., Ltd, Wuhan, China). This aMT6s enzyme-linked immunosorbent assay kits was a competitive immunoassay using an antibody-capture technique with a detection range of 7.813–500 pg/ml and a sensitivity of 4.688 pg/ml. The brief test procedure was as follows. First, 50ul of diluted standard and test sample were pipetted into the wells and 50ul of the biotin-labeled antibody working solution was added, and incubated at 37 °C for 45 min. All wells were washed 3 times. Then 100ul SABA working solution was added and incubated at 37 °C for 30 min. Wash all wells 5 times. Then 90 ul of TMB substrate solution was pipetted and incubated for 30 min at 37 °C. 50ul stop solution was added and the absorbance at 450 nm of each well was immediately measured by a microplate reader. The results were calculated following the manufacturer’s instructions.

### Statistical analysis

All statistical analysis were performed with the IBM SPSS 25.0 program (SPSS Chicago, Illinois, USA). Continuous variables were presented as mean ± standard deviation (SD). The Shapiro–Wilk test was performed to check the normality of the data distributions. For normally distributed variables, use independent sample t-test to compare the two groups of data, otherwise use Mann–Whitney U-test. A Pearson correlation analysis or Spearman correlation analysis was used to assess the associations of melatonin level with CC FD, CT and CVI. Multivariable regression analysis were performed to assess the combined effects of age, AL, spherical equivalent refraction (SER), and aMT6s concentration on CC FD, CT, and CVI. All statistical analysis were performed two-tailed and *P* < 0 0.05 was considered statistically significant.

## Results

### Demographics and clinical characteristics

A total of 25 eyes of 25 night shift medical workers (10 males and 15 females) and 25 eyes of 25 non-night shift workers (12 males and 13 females) were enrolled in this study. There were no significant difference between groups A and group B in sex (*P* = 0.569), age (30.88 ± 1.81 vs. 29.68 ± 3.56 years, *P* = 0.145), IOP (14.67 ± 1.62 vs. 15.44 ± 1.70 mmHg, *P* = 0.108), SER (− 0.46 ± 0.80 vs. − 0.31 ± 0.53 diopters, *P* = 0.437), and AL (23.94 ± 0.73 vs. 23.92 ± 0.72 mm, *P* = 0.918).

### Vessel density of retina and choroid

The vessel density of SCP, DCP, RPC and CC in two groups was reported in Tables 1 and 2. Group A presented significantly higher vessel density of SCP in regions of 2-S (*P* = 0.036) and 3-N (*P* = 0.009), lower vessel density of DCP in region of 2-T (*P* = 0.038), lower vessel density of RPC in region of inside-disk (*P* = 0.031) and higher vessel density of RPC in region of IN (*P* = 0.024) than that of group B. There was no significant difference between groups A and group B in other regions (*P* > 0.05).

**Table 1:** Vessel density of superficial capillary plexus and deep capillary plexus in study population.

	Group A (n = 25)	Group B (n = 25 )	*P*
Vessel density of SCP (%)			
Fovea	9.96 ± 4.22	8.74 ± 5.14	0.184*
2-I	50.58 ± 4.11	48.97 ± 4.51	0.193
2-T	41.41 ± 5.07	39.13 ± 5.05	0.118
2-S	51.41 ± 4.79	48.62 ± 4.33	0.036^†^
2-N	46.41 ± 3.63	44.92 ± 3.24	0.133
3-I	45.04 ± 3.29	43.86 ± 3.33	0.165*
3-T	34.20 ± 5.16	32.24 ± 2.77	0.101
3-S	39.72 ± 4.15	38.37 ± 3.68	0.230
3-N	48.78 ± 4.49	45.72 ± 3.37	0.009^†^
Vessel density of DCP (%)			
Fovea	1.51 ± 1.06	2.32 ± 2.02	0.225*
2-I	6.76 ± 3.47	8.80 ± 4.88	0.218*
2-T	8.06 ± 4.75	10.84 ± 4.47	0.038^†^
2-S	7.37 ± 3.86	8.11 ± 4.19	0.521
2-N	6.97 ± 2.80	7.88 ± 4.96	0.621*
3-I	14.46 ± 5.10	13.38 ± 5.08	0.455
3-T	19.04 ± 7.19	21.20 ± 7.29	0.295
3-S	14.00 ± 5.45	13.14 ± 6.50	0.528*
3-N	11.11 ± 5.97	10.07 ± 6.04	0.544

Data are the means ± standard deviations.

*SCP* superficial capillary plexus, *I* inferior, *T* temporal, *S* superior, *N* nasal, *DCP* deep capillary plexus.

*Mann–Whitney U-test. Others: independent sample t-test.

^†^Statistically significant results (*P* < 0.05).

**Table 2:** Vessel density of radial peripapillary capillary and choriocapillaris in the study population.

	Group A (n = 25)	Group B (n = 25 )	*P*
Vessel density of RPC (%)			
Inside disc	90.20 ± 2.45	91.86 ± 2.80	0.031^†^
NS	53.66 ± 11.76	49.16 ± 8.38	0.126
NI	47.37 ± 11.01	48.69 ± 14.43	0.734*
IN	70.13 ± 7.93	64.36 ± 9.51	0.024^†^
IT	80.12 ± 5.05	79.16 ± 7.42	0.593
TI	49.09 ± 12.49	52.04 ± 15.80	0.421*
TS	51.56 ± 12.10	46.05 ± 16.23	0.179
ST	77.34 ± 5.69	75.11 ± 8.99	0.300
SN	74.34 ± 6.10	72.39 ± 6.89	0.293
Vessel density of CC (%)			
Fovea	72.03 ± 4.70	72.09 ± 6.75	0.973
2-I	69.77 ± 7.37	72.12 ± 5.38	0.204*
2-T	70.88 ± 5.42	70.44 ± 7.23	0.809
2-S	72.77 ± 5.93	72.70 ± 4.99	0.648*
2-N	71.38 ± 5.17	69.62 ± 7.29	0.330
3-I	75.01 ± 3.57	73.93 ± 3.48	0.287
3-T	70.29 ± 8.42	71.10 ± 9.20	0.467*
3-S	72.05 ± 3.66	72.51 ± 4.16	0.678
3-N	73.63 ± 5.15	71.88 ± 5.12	0.235

Data are the means ± standard deviations.

*RPC* radial peripapillary capillary, *NS* nasal superior, *NI* nasal inferior, *IN* inferior nasal, *IT* inferior tempo, *TI* tempo inferior, *TS* tempo superior, *ST* superior tempo, *SN* superior nasal, *CC* choriocapillaris, *I* inferior, *T* temporal, *S* superior, *N* nasal.

*Mann–Whitney U-test. Others: independent sample t-test.

^†^Statistically significant results (*P* < 0.05).

### CC FD size and CC FD density

Compared with group B, group A presented significantly lower CC FD size (32.22 ± 4.6 vs. 25.80 ± 2.01 µm^2^, *P* < 0.001) and CC FD density (8.05 ± 1.93 vs. 6.03 ± 1.44%, *P* < 0.001).

### CT and CVI

Compared with group B, group A presented significantly thicker CT and lower CVI in all regions (all *P* < 0.05) (Table 3).

**Table 3:** Choroidal thickness and choroidal vascularity index in the study population.

	Group A (n = 25)	Group B (n = 25 )	*P*
CT (µm)			
Fovea	436.85 ± 57.67	282.05 ± 56.97	< 0.001^†^
2-I	457.68 ± 75.16	290.44 ± 59.33	< 0.001*^†^
2-T	456.66 ± 59.05	291.32 ± 53.13	< 0.001^†^
2-S	465.06 ± 72.84	281.60 ± 63.47	< 0.001*^†^
2-N	423.63 ± 80.90	251.12 ± 60.23	< 0.001*^†^
3-I	466.44 ± 95.52	302.33 ± 60.30	< 0.001^†^
3-T	461.46 ± 91.19	291.64 ± 44.76	< 0.001^†^
3-S	468.77 ± 109.19	285.70 ± 55.57	< 0.001^†^
3-N	383.27 ± 121.51	198.14 ± 56.01	< 0.001^†^
CVI			
Fovea	0.34 ± 0.05	0.42 ± 0.06	< 0.001*^†^
2-I	0.35 ± 0.04	0.40 ± 0.06	0.001^†^
2-T	0.34 ± 0.04	0.41 ± 0.05	< 0.001^†^
2-S	0.34 ± 0.04	0.42 ± 0.05	< 0.001^†^
2-N	0.37 ± 0.05	0.43 ± 0.06	< 0.001^†^
3-I	0.32 ± 0.02	0.37 ± 0.05	< 0.001^†^
3-T	0.31 ± 0.03	0.37 ± 0.04	< 0.001^†^
3-S	0.32 ± 0.04	0.38 ± 0.04	< 0.001^†^
3-N	0.35 ± 0.04	0.41 ± 0.05	< 0.001^†^

Data are the means ± standard deviations.

*CT* choroidal thickness, *I* inferior, *T* temporal, *S* superior, *N* nasal, *CVI* choroidal vascularity index.

*Mann–Whitney U-test. Others: independent sample t-test.

^†^Statistically significant results (*P* < 0.05).

### Correlation analysis of aMT6s concentration with CC FD, CT and CVI

Group A presented significantly lower aMT6s concentration than that of group B (138.90 ± 74.80 vs. 218.85 ± 78.10 pg/ml, *P* < 0.001). Pearson correlation analysis showed that CC FD size (*P* < 0.05) was significantly correlated with aMT6s concentration and there was no correlation between aMT6s concentration and CC FD density (*P* > 0.05). CT of all regions was negatively correlated with aMT6s concentration (all *P* < 0.05). CVI of the remaining areas was positively correlated with aMT6s (*P* < 0.05) except the regions of 2-I and 2-N (both *P* > 0.05) (Table 4). After adjusting for the effects of age, AL, and SER, multivariable regression analysis showed that aMT6s concentration was still correlated with CC FD size (*P* < 0.05), CT of all regions (all *P* < 0.05), and CVI of the most regions(*P* < 0.05). The age, AL, and SER had no significant effects on CC FD, CT, and CVI (all *P* > 0.05).

**Table 4:** Correlation analysis of 6-sulfatoxymelatonin concentration with choriocapillaris flow deficit, choroidal thickness, and choroidal vascularity index.

	aMT6s	
	*r*	*P*
CC FD size	0.318	0.024^†^
CC FD density	0.147	0.307
CT1	− 0.477	< 0.001^†^
CT2-I	− 0.434	0.002^†^
CT2-T	− 0.454	0.001^†^
CT2-S	-0.432	0.002^†^
CT2-N	-0.437	0.001^†^
CT3-I	-0.424	0.002^†^
CT3-T	− 0.376	0.007^†^
CT3-S	− 0.396	0.004^†^
CT3-N	− 0.459	0.001^†^
CVI1	0.318	0.025^†^
CVI2-I	0.209	0.145
CVI2-T	0.439	0.001^†^
CVI2-S	0.464	0.001^†^
CVI2-N	0.275	0.053
CVI3-I	0.312	0.027^†^
CVI3-T	0.449	0.001^†^
CVI3-S	0.482	< 0.001^†^
CVI3-N	0.400	0.004^†^

*aMT6s* 6-sulfatoxymelatonin, *CC FD*, choriocapillaris flow deficit, *CT* choroidal thickness, *I* inferior, *T* temporal, *S* superior, *N* nasal, *CVI* choroidal vascularity index.

^†^Statistically significant results (*P* < 0.05).

## Discussion

In this study, the retinal and choroidal parameters were quantified and compared between night shift medical workers and non-night shift workers by using SS-OCTA, and the correlation was analyzed between the level of melatonin and above parameters. We found that the circadian rhythm changes caused by night shifts would have an impact on human choroidal microvasculature. The CVI and CC FD decreased and CT thickened in night shift medical workers. In addition, the melatonin level in night shift medical workers decreased and was significantly correlated with choroid parameters.

In this study, compared with group B, the CT was significantly thicker and CVI was significantly lower in group A. CVI was defined as the proportion of the luminal area to the total choroidal area and the decrease of CVI mainly reflected the constriction of choroidal vascularity^16^. The CT was mainly composed of choroidal blood vessels and stroma, and the decrease of CVI suggested that the thickening of CT in our study might be due to choroidal stroma thickening. Choroidal stroma contained numerous non-vascular smooth muscles (NVSMC), which were appeared to affect by both parasympathetic and sympathetic innervation^17^. They could adjust the choroidal stroma thickness by controlling the size of the choroidal lacunae^17^. According to previous study, NVSMC under sympathetic input might stretch, making space for fluid accumulation in the stroma and then leading to interstitial edema, which could made the CT thickening^18^. Night shift workers showed sympathetic excitation^19^, and therefore the CT thickening might be mainly related to choroidal stroma thickening caused by NVSMC extension in this context. In addition, previous study found that the CT in CSC was thickened and both dilation of large choroidal vessels and choroidal interstitial edema played roles in the CT thickening^20^. However, studies have shown that although CT thickening was seen in the uninvolved fellow eyes of CSC patients and no obvious dilation of large choroidal vessels was observed, which indicated that the thickening of choroid stroma might appear earlier than dilation of large choroidal vessels in the pathogenic process in CSC^21^. In this study, the changes of choroid in night shift medical workers were similar to the changes in the uninvolved fellow eyes of CSC mentioned above. Night shift was proved as the independent risk factor of CSC^4^, and these changes in choroid in our study might serve as an biomarker for early high risk CSC detection.

At present, CC images can be obtained through OCTA, which display a granular pattern of bright and dark areas of different sizes. The brighter areas represent higher flow areas, while darker areas are referred to as flow deficits^14^. In our study, compared with group B, group A had lower CC FD, while the two groups had similar vessel density of CC. The inconsistency of the two indicators might related to that the individual capillary in CC was difficult to identify, which made the accurate quantification of CC vessel density challenging^22^. However, CC FD could be accurately calculated from CC images by fuzzy C means algorithm and could indirectly reflect the changes of CC vessel density, which might solve above problems^22^. Recently, CC FD has been widely used as a novel biomarker to reflect the development and activity of various eye diseases, such as diabetic retinopathy and age-related macular degeneration^12,13^. The decrease of CC FD in our study suggested the dilation of CC, which was also be found in the uninvolved fellow eyes of CSC^23^. The similar changes of CC and choroidal stroma between night shift workers and the uninvolved fellow eyes of CSC patients might indicate that the night shifts were related to the early changes of CSC. Previous study has suggested that the choroidal outflow was congested in CSC, and the increase of CC vessel density in the uninvolved fellow eyes of CSC might be a compensatory response to congestion of the outflow, in order to maintain the unobstructed choroidal blood flow^24^. The reason for CC dilation of group A in our study might be similar to the appeal situation. And it could be speculated that the CC dilation in night shift workers might be earlier than the similar changes in the uninvolved fellow eyes of CSC, as in our study, this dilation could only be detected by CC FD which was a more sensitive indicator than CC vessel density. Therefore, CC FD might serve as a more sensitive and accurate indicator to reflect the changes of CC perfusion in high risk CSC.

Melatonin was synthesized and released into the circulation by the pineal gland in a circadian rhythm, peaking at night^25^. Melatonin production was suppressed by light through melanopsin ganglion cells activation in the retina during daylight^26^, which explained the lower level of melatonin in night shift workers exposed to lamps during night. Furthermore, our study found that CC FD size was significantly positively correlated with melatonin. Previous researches showed that the CC FD size was lower during the day and higher at night, and the vessel density of CC was higher in the morning and lower in the evening^11,27^. These changes of CC FD and CC vessel density were match with the trend of melatonin in our study. It was also found that the increase of CT was related to the reduction of melatonin in our study. Previous study showed that the CT was increased in the condition of low melatonin secretion during the day and was decreased in the condition of high melatonin secretion at night, which was consistent with our findings^28^. Besides, it was demonstrated that melatonin could significantly inhibit choroid thickening by reducing the expression of calcium-activated potassium channel KCa2.3 in CSC^9^. A clinical study also showed that oral melatonin could reduce the CT in refractory CSC^29^. Therefore, melatonin might be a potential therapy in eye diseases related to circadian misalignment. And further researches were needed to explore how the melatonin regulated the changes of choroidal microvasculature.

In addition, our study found that the retinal vessel density of night shift medical workers, including SCP, SCP and RPC, did not show significant changes in the vast majority of regions, suggesting that the change of circadian rhythm of night shift medical workers might mainly affect choroidal microvasculature, which was related to the fact that retinal vessels almost unaffected by autonomic innervation, but demonstrated robust autoregulatory capabilities^30^. Further researches were needed to explore retinal microvascular changes over a longer period of time.

There were several limitations in the present study. First, the small sample size of the study might cause some bias in the results and future research with a large sample size will address this issue. In addition, the participants we included were young people, and the effect of circadian rhythm disorder on ocular microcirculation of night shift workers at other ages needs further researches to clarify. Finally, CC FD recently was demonstrated that related with many eye diseases, while the current measurement methods could not fully reflect the real flow deficits of choriocapillaris^22^. Therefore, this limitation need more advanced technologies and algorithms to solve.

To summarize, in our study, we found that night shift medical workers showed thicker CT and lower CVI. This suggested that the thickening of CT in night shift medical workers was mainly related to the thickening of choroidal stroma. In night shift medical workers, CC FD decreased significantly while vessel density of CC had no significant change, indicating that CC FD might be a more sensitive and accurate indicator to reflect the changes of CC perfusion. Our results showed that melatonin was decreased in night shift medical workers and significantly correlated with choroidal parameters. It indicated that the circadian rhythm disorders could affect the secretion of melatonin, and melatonin might affect the choroidal microvasculature through some effects. Further works are needed to determine the mechanism of its action.

## Data Availability

Data will be made available on request.
